# Hyperglycemia affects global 5-methylcytosine and 5-hydroxymethylcytosine in blood genomic DNA through upregulation of *SIRT6* and *TETs*

**DOI:** 10.1186/s13148-019-0660-y

**Published:** 2019-04-15

**Authors:** Er-Feng Yuan, Ying Yang, Lin Cheng, Xujing Deng, Shao-Min Chen, Xin Zhou, Song-Mei Liu

**Affiliations:** 1 Department of Clinical Laboratory, Center for Gene Diagnosis & Program of Clinical Laboratory, Zhongnan Hospital of Wuhan University, 169# Donghu Road, Wuhan, 430071 Hubei Province China; 2 Department of Clinical Laboratory, The Third Affiliated Hospital of Zhengzhou University, Zhengzhou, 450052 Henan Province China; 3 Key Laboratory of Combinatorial Biosynthesis and Drug Discovery, Ministry of Education, School of Pharmaceutical Sciences, Wuhan University, Wuhan, 430071 Hubei Province China

**Keywords:** 5-methylcytosine, 5-hydroxymethylcytosine, *TET*, *SIRT6*, Type 2 diabetes mellitus

## Abstract

**Background:**

Accumulating evidence suggests that epigenetic changes play key roles in the pathogenesis of type 2 diabetes mellitus (T2DM). However, the dynamic regulation of 5-methylcytosine (5mC) and 5-hydroxymethylcytosine (5hmC) in diabetic peripheral blood DNA remains to be elucidated.

**Results:**

We collected fasting blood samples (104 patients and 108 healthy controls) and glucose-stimulated blood samples at different time points (11 patients and 5 healthy controls underwent oral glucose tolerance test (OGTT)), as well as blood samples from six couples of diabetic and control rats. A HPLC-MS/MS system was used for quantifying global 5mC and 5hmC in genomic DNA from white blood cells (WBCs), and qPCR was performed for detecting mRNA expression of *SIRT6* and *TETs*. We found that global 5mC decreased, while global 5hmC increased in both patients and diabetic rats, with lower 5mC being a risk factor of T2DM (OR = 0.524, 95%CI 0.402–0.683, *p* = 1.64 × 10^−6^). The OGTT data from patients showed that 5mC declined within 1 h and then returned to the fasting status at 2 h, while 5hmC rose from 0.5 h to 3 h with increasing glucose. However, the similar patterns were not found in the controls. The mRNA expression of *TET2*, *TET3*, and *SIRT6* was upregulated in patients (*p* = 0.012, *p* = 0.026, and *p* = 0.035, respectively). The similar results were observed in diabetic OGTT and rats. Correlation analysis indicated that *SIRT6* was positively correlated with *TET2* in humans (*r* = 0.277, *p* < 0.001) and rats (*r* = 0.942, *p* < 0.001), in addition to a correlation between glucose and *SIRT6* (*r* = 0.162, *p* = 0.045) and *TET2* (*r* = 0.174, *p* = 0.036).

**Conclusions:**

Hyperglycemia appeared to promote the mRNA expression of *SIRT6* and *TETs*, which in turn might cause the dynamic changes of 5mC and 5hmC in WBCs from T2DM patients.

**Electronic supplementary material:**

The online version of this article (10.1186/s13148-019-0660-y) contains supplementary material, which is available to authorized users.

## Background

With an increasing incidence of obesity, diabetes has become a worldwide epidemic [1], which represents the sixth leading cause of disability, an overwhelming burden on individuals and global healthcare systems [2]. The International Diabetes Federation (IDF) reported that 425 million people were living with diabetes in 2017, with an estimated increase to 629 million by 2045. About > 90% of patients have type 2 diabetes mellitus (T2DM) [2], which is a chronic, complex metabolic syndrome caused by the interaction of genetics, epigenetics, and environmental factors, including obesity, physical inactivity, and aging [3–5]. Relative insulin deficiency resulted from insulin resistance and impaired pancreatic β cell function has been known as the etiology of T2DM [2, 6, 7].

As a well-established epigenetic mark, DNA methylation most often occurs at the 5′-cytosines of CpG dinucleotides [8, 9]. DNA methylation modification is a dynamic process in which methylation could be synthesized de novo, maintained, or removed. DNA methyltransferases (DNMTs) and DNA demethylases corporately maintain these processes with an intricate balance [9, 10]. DNA demethylation can be catalyzed by ten-eleven translocation (TET) enzymes, which mediate the iterative oxidation of 5-methylcytosine (5mC) to 5-hydroxymethylcytosine (5hmC), 5-formylcytosine (5fC), and 5-carboxylcytosine (5caC) [11]. TET proteins are Fe(II) and alpha-ketoglutarate (α-KG)-dependent enzymes, including TET1, TET2, and TET3 [11].

A link has been demonstrated between T2DM and epigenetic alterations [12–14]. Significant changes of TET2 expression and 5hmC abundance have been found in the umbilical veins of gestational diabetes mellitus (GDM) pregnancies [15]. Global 5hmC level in peripheral blood was elevated in poorly glucose controlled T2DM patients with glycated hemoglobin (HbA1c) ≥ 7% (*n* = 25) compared to well-controlled T2DM patients with HbA1c< 7% (*n* = 19) and healthy individuals (*n* = 35) [16]. Recently, some new target genes with altered methylation and expression have been identified in pancreatic islets from T2DM by analyzing DNA methylation of 479,927 CpG sites and the transcriptome, indicating that the aberrant DNA methylation could perturb insulin and glucagon secretion [12]. Some blood-based and age-related DNA methylation biomarkers have also been identified in human islets and were found to be associated with insulin secretion and diabetes [4]. Data from retinal capillary cells and diabetic mouse models have shown that hyperglycemia leads to *MMP-9* promoter hypomethylated and the activation of the demethylation machinery, resulting in its increased transcription. Silencing of TET2 could prevent hyperglycemia-induced increase in 5hmC and MMP-9 transcription [17]. A zebrafish study has further confirmed that hyperglycemia activates TETs to induce the demethylation of cytosines throughout the genome [18]. On the contrary, a recent study reported that hyperglycemic conditions had an adverse effect on the DNA 5-hydroxymethylome via the destabilization of TET2 [19].

SIRT6, a member of the sirtuin protein family, has been reported to mediate many important cellular processes, such as DNA repair, maintenance of genomic stability, anti-inflammation, gluconeogenesis, and insulin secretion [20–26].

Although studies have revealed altered DNA methylation patterns in pancreatic islets, adipose tissue, and skeletal muscle [5, 13, 27–30], it remains poorly understood that the dynamic regulation of 5hmC in peripheral blood-derived genomic DNA from T2DM patients, and whether blood glucose and the histone deacetylase SIRT6 are involved in this process. Therefore, we used a HPLC-MS/MS system for quantifying 5mC and 5hmC in genomic DNA from white blood cells (WBCs) in humans and rats and qPCR for detecting mRNA expression of *SIRT6* and *TETs* to test the hypothesis that there might exist a link across SIRT6, TETs, and 5hmC in T2DM.

## Results

### Clinical characteristics of the study subjects

The clinical characteristics of 104 T2DM patients and 108 controls are shown in Table 1. There were no significant differences in age and gender between T2DM patients and controls (*p* = 0.301 and *p* = 0.567, respectively). As expected, patients had higher levels of fasting blood glucose (*p* < 0.001), TC (*p* = 0.028), TG (*p* < 0.001), and LDL-C (*p* = 0.014) and lower HDL-C (*p* < 0.001) than those of the controls. Pearson correlation analysis indicated that glucose was correlated with TG, TC, HDL-C, LDL-C and HbA1c (Additional file 1: Table S1). More than half of the patients (*n* = 68, 65.4%) also suffered from macrovascular diseases. The average of HbA1c in patients was significantly above the reference range (8.56% vs < 6.5%).

**Table 1:** Clinical characteristics of the study subjects

	T2DM patients (*n* = 104)	Controls (*n* = 108)	*p* value
Age (years)	59.19 ± 11.88	57.59 ± 10.60	0.301^a^
Gender (male/female)	58/46	56/52	0.567^b^
Fasting blood glucose (mmol/L)	8.58 ± 0.49	4.81 ± 0.05	< 0.001^a^
Total cholesterol (TC, mmol/L)	4.47 ± 0.83	4.25 ± 0.52	0.028^a^
Triglyceride (TG, mmol/L)	2.31 ± 1.93	1.09 ± 0.32	< 0.001^a^
High-density lipoprotein cholesterol (HDL-C, mmol/L)	1.19 ± 0.33	1.53 ± 0.30	< 0.001^a^
Low-density lipoprotein cholesterol (LDL-C, mmol/L)	2.80 ± 0.80	2.57 ± 0.40	0.014^a^
Insulin (uU/mL)	10.12 ± 6.85		
HbA1c (%)	8.56 ± 2.22		
Vascular ultrasound			
With macrovascular diseases	68 (65.4%)		
Without macrovascular diseases	28 (26.9%)		
N/A	8 (7.7%)		

Data are presented as mean ± SD

*N/A* not available

^a^Student’s *t* test

^b^*χ*^2^ test

### Decreased 5mC and increased 5hmC in T2DM patients

**Fig. 1:**
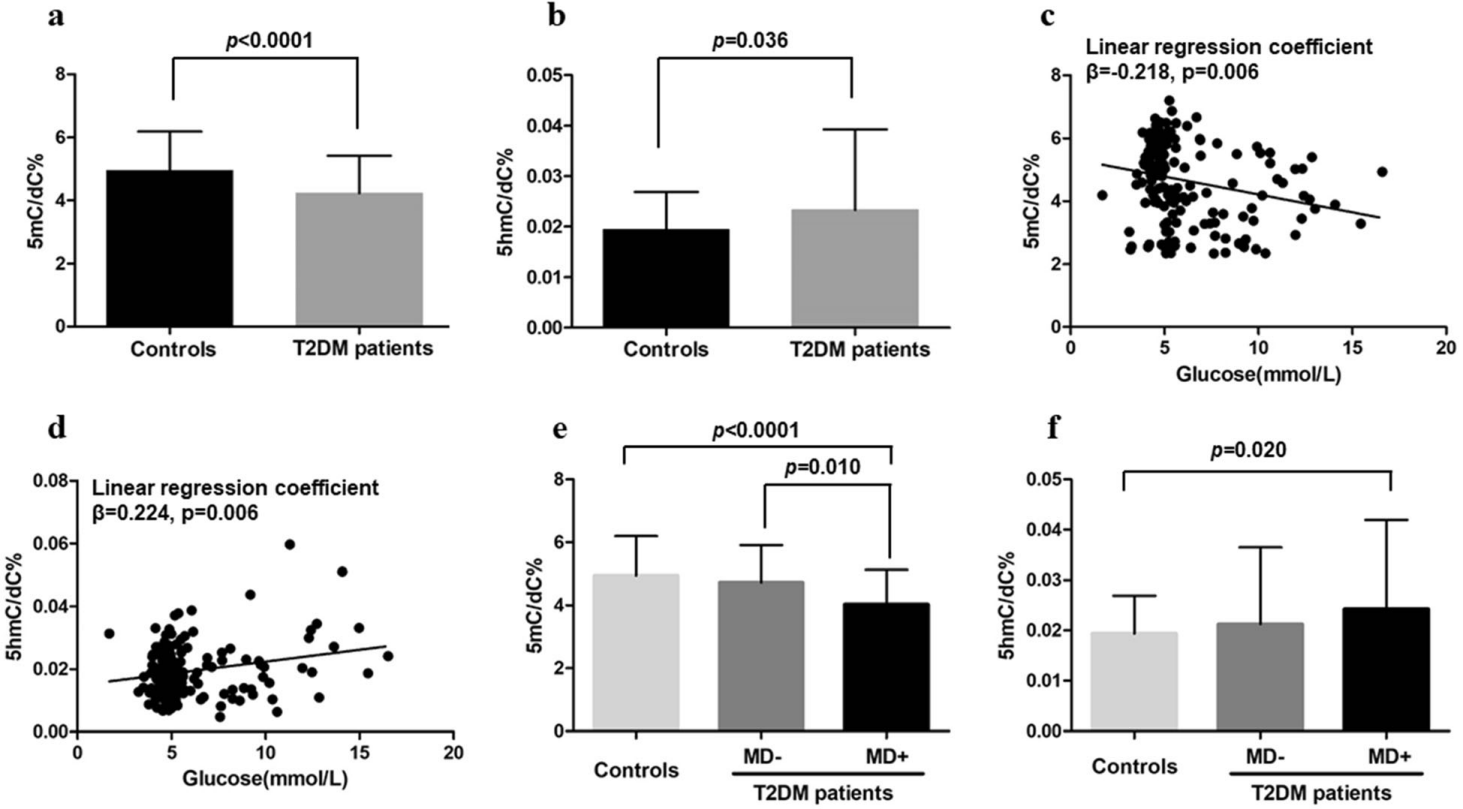
Decreased 5mC and increased 5hmC in T2DM patients. **a** 5mC and **b** 5hmC contents in T2DM patients and controls. Linear regression analysis between glucose and **c** 5mC and **d** 5hmC in all subjects after controlling age and gender. **e** 5mC and (**f**) 5hmC contents in T2DM patients with (MD+) or without (MD−) macrovascular diseases and controls. 5mC and 5hmC contents were detected by HPLC-MS/MS, and data are expressed as the means ± SD

We first detected the global 5mC and 5hmC contents in 212 human DNA samples by HPLC-MS/MS in MRM mode. The 5mC contents in T2DM patients were significantly lower than those of the controls (4.23 ± 1.19% vs 4.95 ± 1.24%, *p* < 0.0001; Fig. 1a), while the 5hmC contents were elevated (0.0233 ± 0.0169% vs 0.0194 ± 0.0075%, *p* = 0.036; Fig. 1b). Significant correlations were found between the fasting blood glucose and 5mC (*β* = − 0.218, *p* = 0.006; Fig. 1c) and 5hmC (*β* = 0.224, *p* = 0.006; Fig. 1d) after controlling age and gender.

We further performed a multivariate logistic regression adjusted for age and gender to explore if the altered 5mC and 5hmC were the risk factors of T2DM. The results showed that lower 5mC was a risk factor of T2DM (OR = 0.524, 95%CI 0.402–0.683, *p* = 1.64 × 10^−6^); however, the 5hmC was not (*p* = 0.162).

Next, we evaluated the difference of 5mC and 5hmC between T2DM patients with or without macrovascular diseases and controls. The global 5mC level was the lowest in T2DM with macrovascular diseases, which is significantly lower than the controls (*p* < 0.0001) and those without macrovascular diseases (*p* = 0.010, Fig. 1e). Interestingly, although the contents of 5hmC were elevated in T2DM with macrovascular diseases compared with controls (*p* = 0.020), the difference of 5hmC between T2DM with and without macrovascular diseases was not statistically significant (Fig. 1f).

### Dynamic 5mC and 5hmC mediated by glucose stimulation

**Fig. 2:**
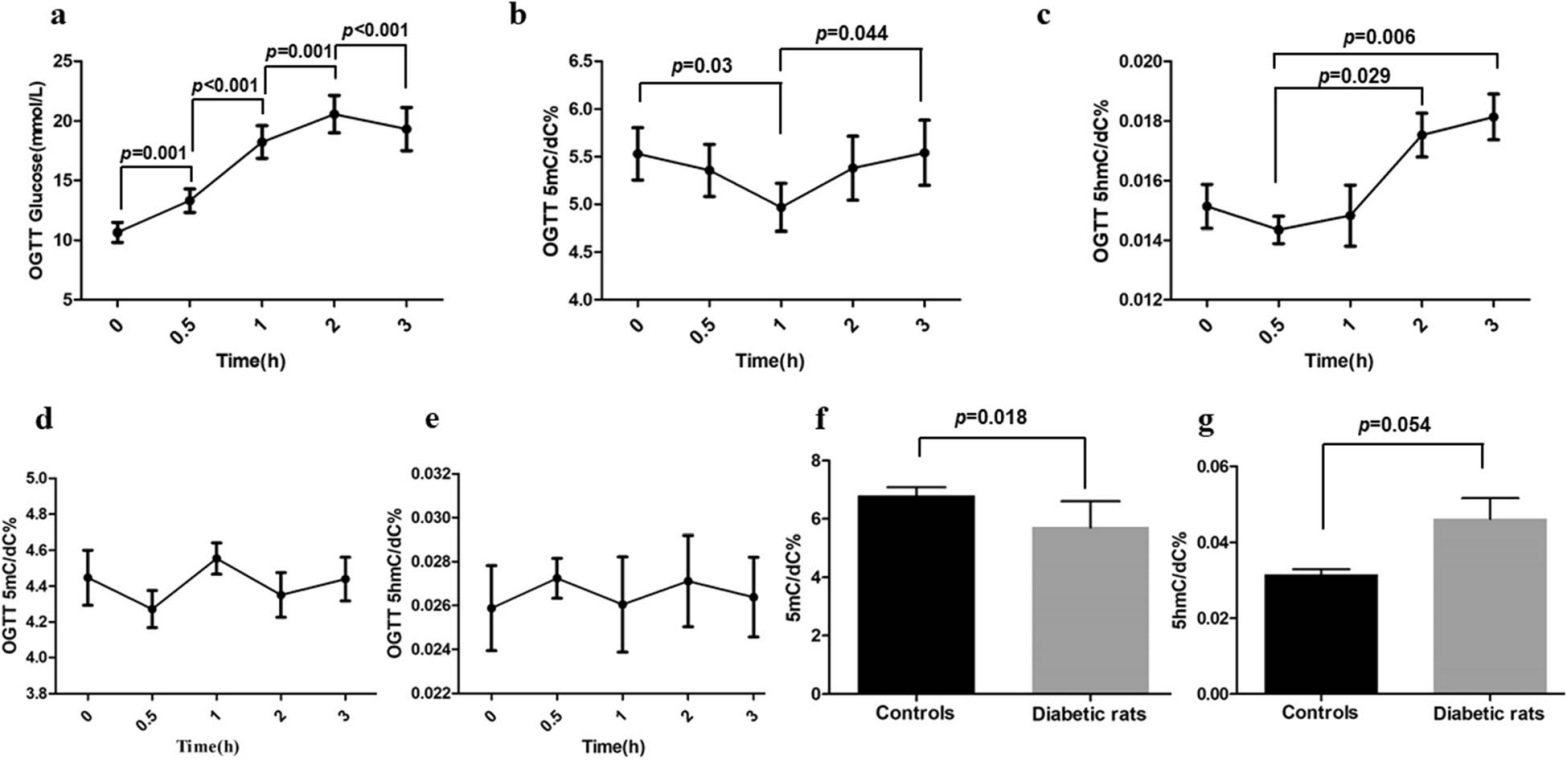
Dynamic 5mC and 5hmC mediated by glucose stimulation. The levels of **a** glucose, **b** 5mC, and **c** 5hmC at five different time points (0, 30, 60, 120, and 180 min) of OGTT from diabetes patients. The levels of **d** 5mC and **e** 5hmC at five different time points (0, 30, 60, 120, and 180 min) of OGTT from controls. **f** 5mC and **g** 5hmC contents in diabetic rats and controls. The contents of 5mC and 5hmC were detected by HPLC-MS/MS, and data are expressed as the means ± SD

To further elucidate the role of glucose in the dynamic regulation of 5mC and 5hmC, we performed an oral glucose tolerance test (OGTT) with a standard dose of 75 g glucose in 11 T2DM patients and 5 healthy controls. Peripheral blood samples were collected before glucose intake (fasting) and 30, 60, 120, and 180 min after glucose load. The contents of 5mC and 5hmC at five different time points were then analyzed with HPLC-MS/MS. As illustrated in Fig. 2, with increasing blood glucose (Fig. 2a), 5mC levels declined within 1 h and then went back to fasting status at the time point of 2 h; the difference of 5mC is significant between fasting and 1 h (*p* = 0.030) and 1 h and 3 h (*p* = 0.044) in T2DM patients (Fig. 2b). In contrast, 5hmC levels rose from 0.5 h to 3 h with increasing blood glucose, the difference of 5hmC was significant between 0.5 h and 3 h (*p* = 0.006, Fig. 2c) in T2DM patients. However, the similar patterns were not found in the controls. (Fig. 2d, e).

Similar to the results from human blood, global 5mC contents in diabetic rats were significantly decreased compared with controls (*p* = 0.018, Fig. 2f), while 5hmC contents slightly increased, with marginal significance (*p* = 0.054; Fig. 2g).

### Upregulation of *TET*s and *SIRT6* in both T2DM patients and diabetic rats

**Fig. 3:**
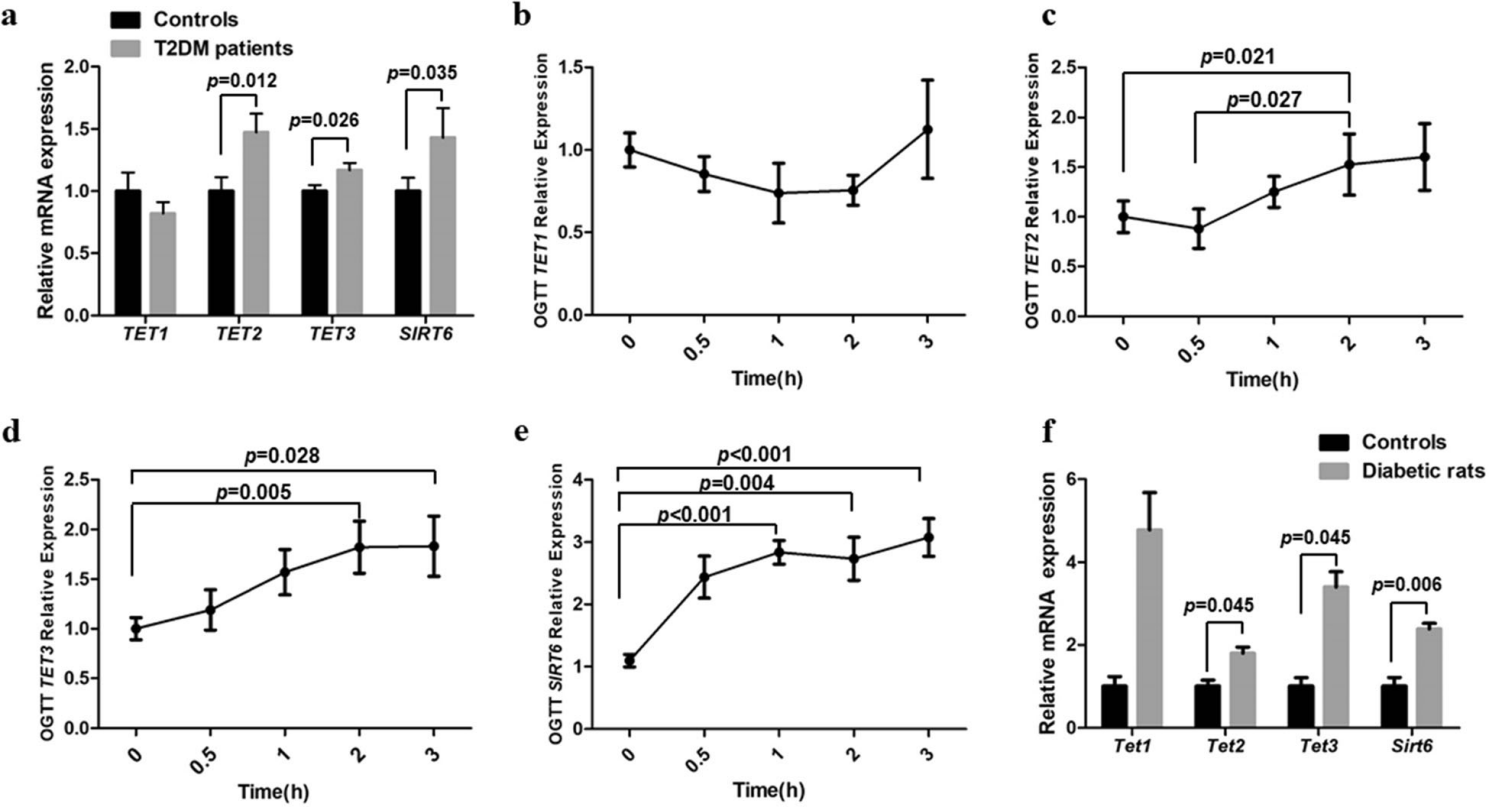
Upregulation of *TETs* and *SIRT6* in both T2DM patients and diabetic rats. **a** The mRNA expression of *TETs* and *SIRT6* in T2DM patients and controls. **b**–**e** The mRNA expression of *TETs* and *SIRT6* at five different time points (0, 30, 60, 120, and 180 min) of OGTT. **f** The mRNA expression of *TETs* and *SIRT6* in diabetic rats and controls. The mRNA expression levels were examined by qPCR and normalized to *GAPDH* and *β-ACTIN*, and data are expressed as the means ± SEM

To explore the possible reasons for decreased 5mC and increased 5hmC in diabetes, we utilized qPCR to determine the mRNA expression of demethylation machinery genes and *SIRT6* in diabetic patients and rats. Student’s *t* test showed that the mRNA expression levels of *TET2*, *TET3*, and *SIRT6* were 1.47-, 1.17-, and 1.43-fold higher in T2DM patients than that in the controls (*p* = 0.012, *p* = 0.026, and *p* = 0.035, respectively) (Fig. 3a). However, there was no significant difference in *TET1* mRNA expression (*p* = 0.302) between T2DM patients and controls (Fig. 3a).

Except for *TET1* (Fig. 3b), the mRNA expression of *TET2*, *TET3*, and *SIRT6* significantly increased after glucose stimulation in 11 cases of T2DM patients participating OGTT. The paired Student *t* test indicated that *TET2* mRNA expression significantly elevated at 2 h when compared with fasting status and 0.5 h (2 h vs fasting, *p* = 0.021; 2 h vs 0.5 h, *p* = 0.027; Fig. 3c); *TET3* mRNA upregulated at 2 h and 3 h (2 h vs fasting, *p* = 0.005; 3 h vs fasting, *p* = 0.028; Fig. 3d); and *SIRT6* mRNA levels also showed an increase from 1 h after glucose intake (1 h vs fasting, *p* < 0.001; 2 h vs fasting, *p* = 0.004; 3 h vs fasting, *p* < 0.001; Fig. 3e).

To confirm the observations from human samples, we further test the mRNA levels of *Tet1*, *Tet2*, *Tet3*, and *Sirt6* in blood samples from six couples of diabetic and control rats. Consistent with the results from T2DM patients, the mRNA expressions of *Tet2* (*p* = 0.045), *Tet3* (*p* = 0.045), and *Sirt6* (*p* = 0.006) were significantly increased in diabetic rats compared with the controls (Fig. 3f). However, there was no significant difference in *Tet1* mRNA levels between diabetic rats and the controls (*p* = 0.285) (Fig. 3f).

### Positive association of *SIRT6* expression with *TET2* and glucose

**Fig. 4:**
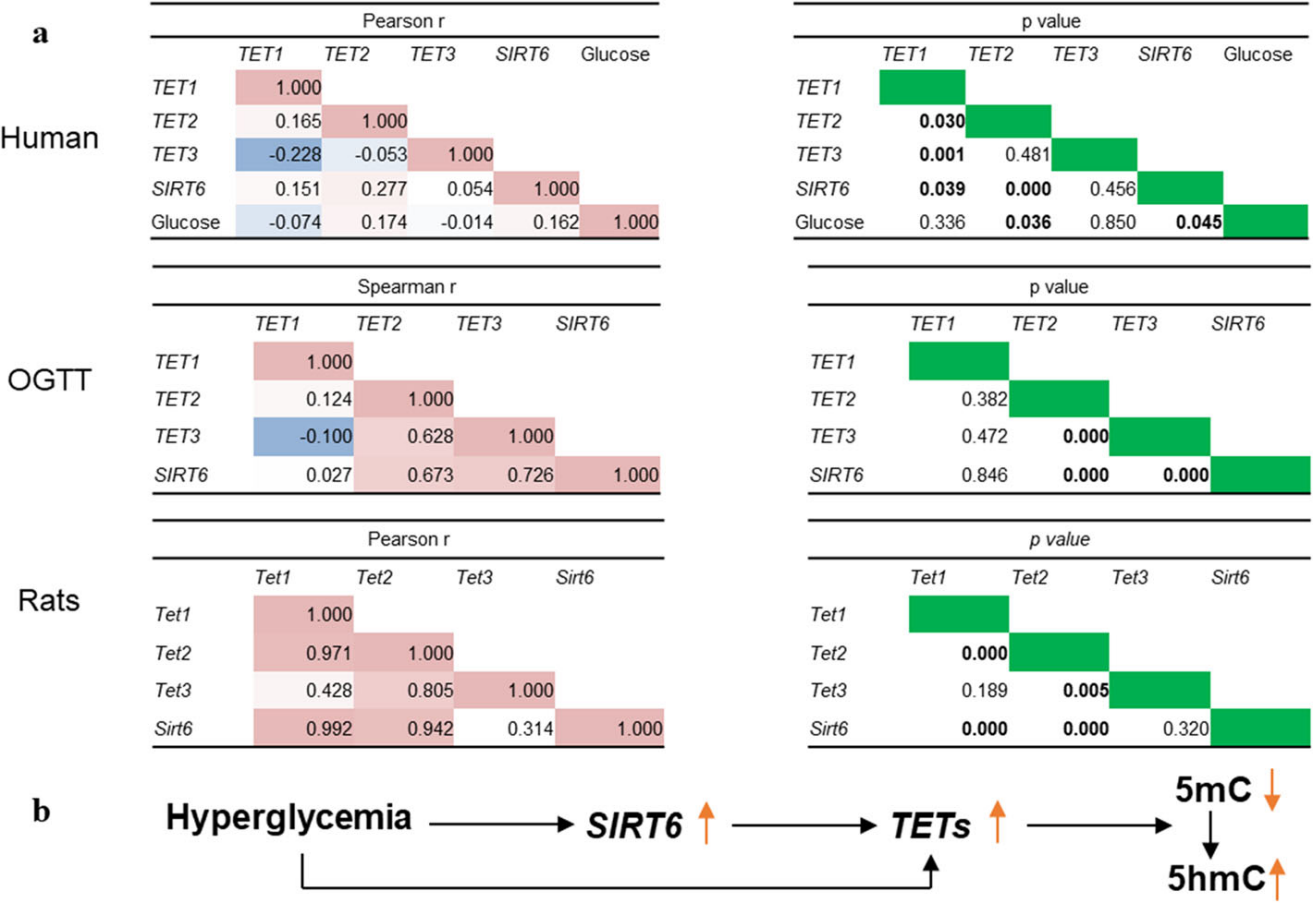
The correlations cross *SIRT6, TETs*, glucose and the possible work model. **a** Correlation analysis between mRNA expression of *SIRT6*, *TETs*, and glucose in all human subjects (upper panel), OGTT (middle panel), and rats (lower panel). The correlation coefficients (*r*) (left panel) and *p* values (right panel) are shown. **b** The possible work model of DNA demethylation promoted by hyperglycemia via *SIRT6* and *TETs*

Figure 4 presents the correlations across *TETs*, *SIRT6*, and glucose. In T2DM patients and controls, the Pearson correlation analysis revealed that glucose was positively associated with *TET2* (*r* = 0.174, *p* = 0.036) and *SIRT6* (*r* = 0.162, *p* = 0.045), while *SIRT6* was positively associated with *TET1* (*r* = 0.151, *p* = 0.039) and *TET2* (*r* = 0.277, *p* < 0.001). The Spearman correlation analysis based on the results of OGTT showed that *SIRT6* positively correlated with *TET2* (*r* = 0.673, *p* < 0.001) and *TET3* (*r* = 0.726, *p* < 0.001). In rats, the Pearson correlation analysis revealed that *SIRT6* was positively associated with *TET1* (*r* = 0.992, *p* < 0.001) and *TET2* (*r* = 0.942, *p* < 0.001). Notably, the association of *TET2 with SIRT6* existed in the studies of case - control, OGTT, and animals.

## Discussion

The incidence and prevalence of T2DM have been on the rise globally. China definitely ranks first in terms of the absolute number of diabetic patients. The importance of epigenetics in T2DM has not been fully stated. Here, HPLC-MS/MS was used to measure global 5mC and 5hmC contents in T2DM blood samples. Our results demonstrated that 5mC decrease and 5hmC increase were mediated by glucose in T2DM patients and diabetic rats through upregulation of *SIRT6* and *TETs*.

Indeed, the results would be more informative if we analyzed 5mC and 5hmC levels in different compositions of blood. However, isolation of cell subsets to detect 5mC and 5hmC needs ~ 10 mL fresh peripheral blood as previously reported [31]. Here, we used a more clinically feasible amount of blood sample (2 mL) to perform experiments, which is not enough to isolate different composition. To exclude the differences between diabetic and non-diabetic individuals is the effect of differing blood cell proportions, we compared the differences of WBCs, neutrophils, lymphocytes, and monocytes between T2DM patients and controls. The results showed that there were no significant differences in different blood cells between these two groups (Additional file 1: Figure S1).

Despite some reports suggesting alterations in 5mC in human islets from T2DM [4, 12, 32], 5hmC in umbilical vein tissues from GDM by genome-wide profiling [15], as well as controversial changes in 5mC and 5hmC in peripheral blood from diabetic patients in two independent studies with commercial kits or HPLC-MS/MS [16, 19]. A study with human peripheral blood in Colombia by *Pinzon-Cortes JA.* et al. reported that both 5mC and 5hmC were increased in poorly glucose controlled diabetic patients with HbA1c > 7% [16], while another recent study found that 5hmC in peripheral blood monocytes from diabetic patients (*n* = 18) was lower than the healthy controls (*n* = 15) [19]. Consistent with a previous report [16], our findings also suggested that 5hmC in WBCs fromT2DM patients statistically increased independent of macrovascular events, and demethylation machinery genes *TET2* and *TET3* concordantly upregulated in patients and animals, which might attribute to the elevation of 5hmC. With this data, we provided further evidence of the higher content of 5hmC in T2DM. Because 5mC is required as a substrate for oxidation to generate 5hmC by TETs, the increase in 5hmC could be partially responsible for the reduced 5mC. In line with a recent study [19], our patients without macrovascular complications had the similar 5mC levels to the controls.

Given the importance of SIRT6 in DNA methylation, insulin secretion, as well as glucose and fat metabolism [6, 20–26, 33], we tested mRNA expression of *SIRT6* and found that *SIRT6* elevated significantly in both patients and rats. Similarly, loss of *SIRT6* could induce global hypomethylation, hypoglycemia, and increased fat deposition in hepatocellular carcinoma [33]. *Sirt6* knockdown in MIN6 beta cells led to a significant decrease in glucose-stimulated insulin secretion [6] and an increase in palmitate-induced pancreatic β-cell dysfunction and apoptosis [20]. Interestingly, a recent in vivo pharmacological study has documented that Sirt6 inhibition improved glucose tolerance in the T2DM mouse model, associated with reduced insulin, triglycerides, and cholesterol levels in plasma, suggesting that SIRT6 inhibitor might be a strategy for glycemic control in T2DM [34]. In contrast, embryonic stem cells (ESCs) derived from *Sirt6* knockout mice showed an upregulation of Tet enzymes and elevated production of 5hmC [35].

Clearly, hyperglycemia is known as a distinct pathological feature of T2DM; intensive control of glucose would be beneficial for preventing the progress of T2DM [8]. Thus, we investigated the correlations between glucose and 5mC and 5hmC. To our knowledge, the current work is the first study to reveal the role of glucose in the regulation of dynamic DNA methylation in the human body. Our findings in general populations suggested glucose negatively correlated with 5mC and were positively associated with 5hmC. Furthermore, the dynamic changes of 5mC and 5hmC contents after oral glucose were in line with what we found in T2DM patients and diabetic rats exposed to prolonged hyperglycemia, making the altered DNA methylation pattern in blood from T2DM patients likely to be caused of persistent hyperglycemia.

To further illustrate how high glucose might lead to DNA methylation alterations, we tested the mRNA expression of *SIRT6* and *TETs* in all study subjects and rats. Our results suggested that *SIRT6* positively correlated with *TET2* in humans and rats, and there existed a link between glucose and *SIRT6* and *TET2*. A possible model in T2DM patients might work as Fig. 4b, in which hyperglycemia could promote DNA demethylation via upregulation of *SIRT6* and *TET2*.

We realized there were several limitations in the current study. Firstly, the sample size of the OGTT was small. Secondly, using peripheral blood did not allow us to detect the differences of 5mC and 5hmC between diabetic and non-diabetic individuals in different blood cell components. Although this study only detected the mRNA expression of interest genes due to the limited blood volume, to our knowledge, we are the first to report the alterations of 5mC, 5hmC, *TETs*, and *SIRT6* at different time points after oral glucose uptake in diabetic patients.

## Conclusion

In summary, our results reflected the dynamic DNA methylation influenced by glucose stimulation in human blood, suggesting that epigenetic alterations including DNA methylation emerged as an important determinant of diabetes, which further verified our hypothesis and highlighted that SIRT6 might be a potential target for glucose control.

## Methods

### Human blood samples

Our study was approved by the Medical Ethics Committee of Zhongnan Hospital of Wuhan University (approval number 2014039), and written informed consents were obtained from each participant. A 1:1 matched case-control samples were collected (T2DM patients = 104, controls = 108) from Zhongnan Hospital of Wuhan University. Patients were diagnosed as T2DM according to the Standards of Medical Care in Diabetes 2012 of the American Diabetes Association (ADA). The age- and gender-matched controls were randomly enrolled from healthy individuals who were free of endocrine diseases, cardiovascular diseases, and cancers. Another 11 T2DM patients and five controls participating in OGTT were also enrolled for further investigation of the dynamic regulation of 5mC and 5hmC after glucose stimulation. Biochemical parameters were determined by Beckman AU5800 chemistry analyzer with Beckman commercial kits in the clinical laboratory, including fasting blood glucose, triglycerides (TG), total cholesterol (TC), high-density lipoprotein cholesterol (HDL-C), and low-density lipoprotein cholesterol (LDL-C). Blood HbA1c was detected by ADAMS HA-8160 high-performance liquid chromatography. Serum insulin was determined by Abbott i4000SR Immunology Analyzer with i2000 commercial kits. Artery ultrasound was utilized to test the presence of atherosclerotic plaque, an indicator for macrovascular diseases.

### Diabetic animal model establishment

Male Wistar rats (6–8 weeks old; 175–200 g) were purchased from the Hubei Provincial Center for Disease Control and Prevention. Diabetic rats were established according to our previous protocol [36]. Rats that had blood glucose levels higher than 11.6 mmol/L were regarded as diabetic rats. Six diabetic and six non-diabetic rats were used for this study. All animal procedures were performed in accordance with the guidelines of the Institutional Animal Care and Use Committee of Wuhan University.

### DNA extraction and enzymatic digestion

Genomic DNA was extracted from peripheral WBCs. DNA digestion was performed according to the previous study with minor modification [37]. Briefly, ~ 1.5 μg DNA in 8.5 μL ddH_2_O was denatured at 95 °C for 5 min and chilled on ice for 2 min. Then, 1 μL of S1 nuclease buffer and 100 units (0.5 μL) S1 nuclease (Thermo Scientific, Waltham, MA) were added and incubated at 37 °C for 4 h. Next, 24.5 μL H_2_O, 4 μL alkaline phosphatase buffer, 15 units (0.5 μL) of alkaline phosphatase (TaKaRa, Dalian, China), and 0.001 units (1 μL) of venom phosphodiesterase I were added to the solution (Sigma, St. Louis, MO). The mixture (40 μL) was incubated for another 2 h at 37 °C. The resulting solution was extracted with an equal volume of chloroform twice and followed by an ultrafiltration with YM-10 column (Microcon, Shanghai, China). The flow-through was monitored by HPLC-MS/MS to quantify the content of 5mC and 5hmC.

### Measurement of global 5mC and 5hmC

Analysis of 5mC and 5hmC was operated on an Agilent HPLC-MS/MS 6410 Triple Quad mass spectrometer (Agilent, CA) with an electrospray ionization source under positive ion mode. An ultimate XB-C18 column (150 mm × 2.1 mm i.d., 3 μm, Weltech Co., Ltd., Wuhan, China) was used for the separation of target analytes. The parameters for mass spectrometry detection were as follows: gas flow, 10 L/min; nebulizer pressure, 40 psi; drying gas temperature, 320 °C; and capillary voltage, 3500 V. The product ions derived from the precursor ions were monitored by multiple reaction monitoring mode (MRM) using the mass transitions (precursor ions → product ions) of 2′-deoxyadenosine (dA) (252.4 → 136.2), thymidine (T) (243.3 → 127.2), 2′-deoxyguanosine (dG) (268.4 → 152.4), 2′-deoxycytidine (dC) (228.4 → 112.2), 5mC (242.0 → 126.0), and 5hmC (258.0 → 142.0).

The linearity was examined using 2.5 pmol dC standard supplemented with 5mC and 5hmC at different amounts ranging from 0.05 pmol to 0.5 pmol and 0 pmol to 0.005 pmol, respectively. The calibration curves were plotted with the expected molar ratio of 5mC/dC or 5hmC/dC vs the observed molar ratio of 5mC/dC or 5hmC/dC based on data obtained from triplicate measurements. The results showed linearity within the range of 2–20% (molar ratio of 5mC/dC) and 0–0.2% (molar ratio of 5hmC/dC) with a coefficient of determination (*R*^2^) higher than 0.99.

### Quantification of mRNA expression by qPCR

To assess the mRNA expression of human *TET1*, *TET2*, *TET3*, and *SIRT6,* and rat *Tet1*, *Tet2*, *Tet3*, *Sirt6*, total RNA was extracted from the peripheral WBCs using TRIzol reagent (Invitrogen, Carlsbad, CA), then, cDNA synthesis was performed with DNase treatment and reverse transcription (TOYOBO, Osaka, Japan). The mRNA expression of target genes and the reference genes *GAPDH* and *β-ACTIN* were measured by quantitative qPCR using CFX96 Touch TM Real-Time PCR System with iTaq TM Universal SYBR Green Supermix (Bio-Rad, Hercules, CA). Intron-spanning primer sequences are listed in Additional file 1: Table S2, and the results are displayed as the means ± SEM.

### Statistical analysis

We performed all the statistical analyses using SPSS 21.0 software. A normality test was used to explore the data distribution. Student *t* test was used to evaluate the differences for normally distributed data between two groups. A paired Student *t* test was used to compare the differences between different time points of OGTT. A logistic regression model adjusted for age and gender was performed to explore if the altered 5mC and 5hmC were the risk factors of T2DM. A linear regression model adjusted for age and gender was used to explore the correlations between glucose and 5mC and 5hmC. Pearson correlation coefficients for normal distributed data and Spearman correlation coefficients for non-normal distributed data were used for evaluation of the correlations between gene expression and 5mC and 5hmC. *p* < 0.05 was considered to be statistically significant.

## Additional file

Additional file 1: **Figure S1.** The comparison of white blood cells (WBC), neutrophil (NEUT), lymphocyte (LYMPH) and monocyte (MONO) in T2DM patients and controls. Data are expressed as the means ± SD. **Table S1.** Correlations between glucose and biochemical parameters. **Table S2.** Primers used for qPCR. (DOCX 64 kb)

## Abbreviations

5hmC: 5-hydroxymethylcytosine
5mC: 5-hethylcytosine
HbA1c: Glycated hemoglobin
HDL-C: High-density lipoprotein cholesterol
HPLC-MS/MS: High-performance liquid chromatography-mass spectrometry
LDL-C: Low density lipoprotein cholesterol
OGTT: Oral glucose tolerance test
SIRT6: Sirtuin 6
TC: Total cholesterol
TETs: Ten-eleven translocation proteins
TG: Triglycerides
WBC: White blood cell
