# Supplementary material for: Hyperglycemia affects global 5-methylcytosine and 5-hydroxymethylcytosine in blood genomic DNA through upregulation of SIRT6 and TETs
Source: Clin Epigenetics. 2019 Apr 15;11:63. doi: 10.1186/s13148-019-0660-y (PMC6466651; doi:10.1186/s13148-019-0660-y)
Supplement: Supplementary file 1 — Figure S1. The comparison of white blood cells (WBC), neutrophil (NEUT), lymphocyte (LYMPH) and monocyte (MONO) in T2DM patients and controls. Data are expressed as the means ± SD. Table S1. Correlations between glucose and biochemical parameters. Table S2. Primers used for qPCR. (DOCX 64 kb) [file 13148_2019_660_MOESM1_ESM.docx]

**
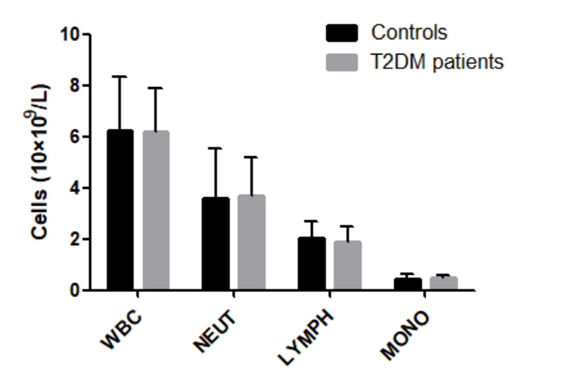
Figure S1.** The comparison of white blood cells (WBC), neutrophil (NEUT), lymphocyte (LYMPH) and monocyte (MONO) in T2DM patients and controls. Data are expressed as the means ± SD.

**Table S1. Correlations between glucose and biochemical parameters**

|  | Fasting blood glucose (mmol/L) | |
| --- | --- | --- |
|  | Pearson correlation coefficient | *p* |
| Total cholesterol (TC, mmol/L) | 0.191 | 0.011 |
| Triglyceride (TG, mmol/L) | 0.310 | 2.47**×**10^-5^ |
| High-density lipoprotein cholesterol (HDL-C, mmol/L) | -0.366 | 1.32**×**10^-6^ |
| Low-density lipoprotein cholesterol (LDL-C, mmol/L) | 0.218 | 0.005 |
| HbA1c (%) | 0.702 | 8.09×10^-13^ |
| Insulin (uU/mL) | -0.148 | 0.247 |

**Table S2. Primers used for qPCR.**

| **Genes** | **Primers (5’ →3’)** |
| --- | --- |
| **Human** | |
| *GAPDH* | Forward: TCTATAAATTGAGCCCGCAGC  Reverse: CCAATACGACCAAATCCGTTG |
| *β-ACTIN* | Forward: CCAGCTCCTCCCTGGAGAAG  Reverse: ACAGGACTCCATGCCCAGG |
| *TET1* | Forward: GCGACCCTTGGTGCTAAACC  Reverse: CAGGGCCTCACCATGAACTG |
| *TET2* | Forward: GCTTCCATTCTGGAGCTTTG  Reverse: GGACATGATCCAGGAAGAGC |
| *TET3* | Forward: CCATTCAGGACCCCGAGAAC  Reverse: CCACTGAGGGTGGGTGTGAG |
| *SIRT6* | Forward: AGGATGTCGGTGAATTACGC  Reverse: CCAGTTCCCACACCTTCC |
| **Rat** | |
| *Gapdh* | Forward: ACCTTTGATGCTGGGGCTGGC  Reverse: GGGCTGAGTTGGGATGGGGACT |
| *β-actin* | Forward: CGCGTCCACCCGCGAG  Reverse: CCTGGTGCCTAGGGCG |
| *Tet1* | Forward: GCCAACCAGGAAGAGGCGACTG  Reverse: GAGGAAGCCTGCAGGGGACAG |
| *Tet2* | Forward: GCCTTCGGATTCAGACAC  Reverse: ACCATTCGGTTGGAGTTA |
| *Tet3* | Forward: CCCCTACAGTATGAGCAGCG |
|  | Reverse: TAGAGCATGGAGGTCTGGCT |
| *Sirt6* | Forward: CCGTCTGGTCATTGTCAACC |
|  | Reverse: GCAGCCTCTGTCTTCACTCTT |
